# Supplementary material for: Songbirds work around computational complexity by learning song vocabulary independently of sequence
Source: Nat Commun. 2017 Nov 1;8:1247. doi: 10.1038/s41467-017-01436-0 (PMC5663719; doi:10.1038/s41467-017-01436-0)
Supplement: Supplementary file 1 — Supplementary information [file 41467_2017_1436_MOESM1_ESM.pdf]

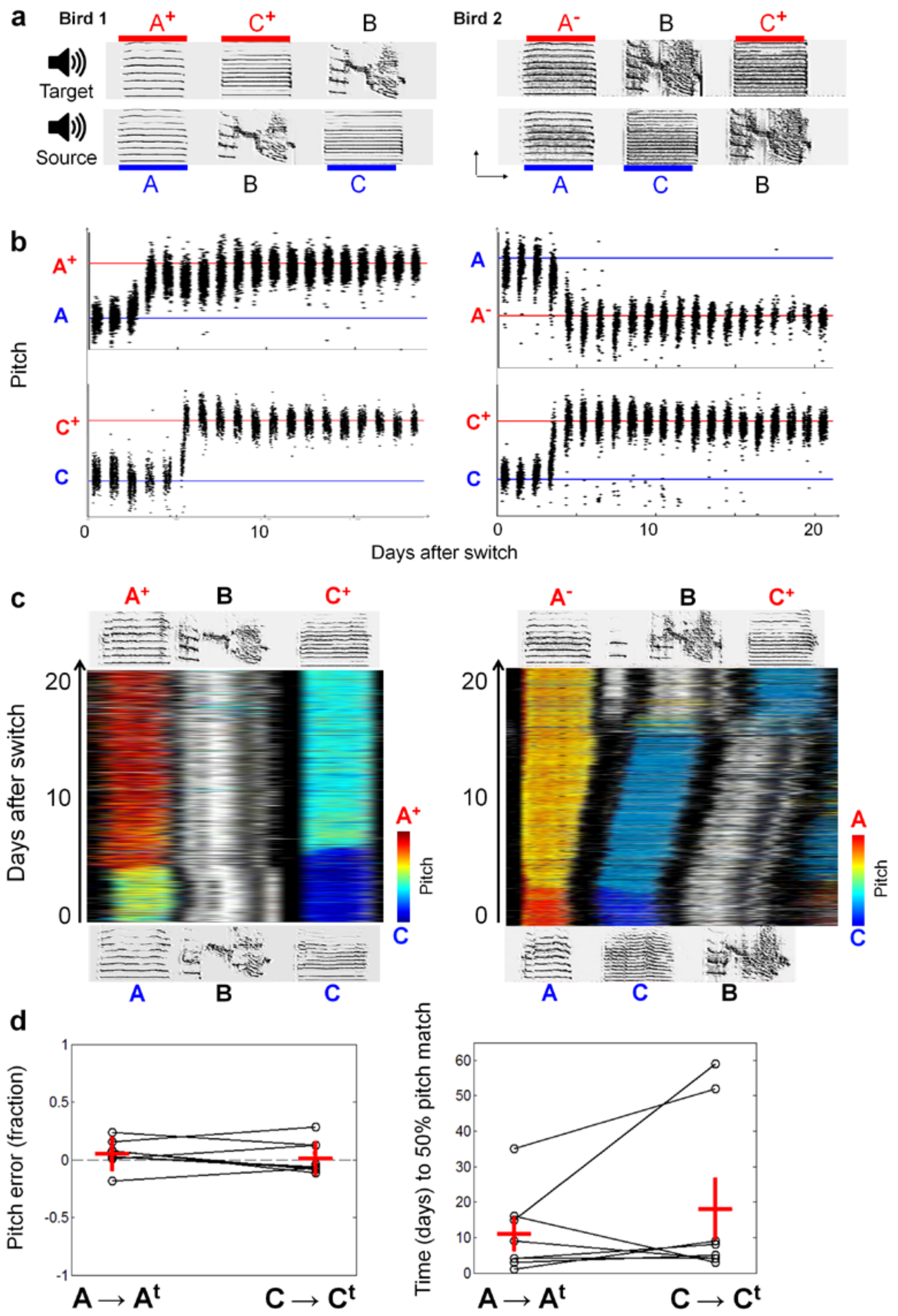

Supplementary Figure 1

Pitch error corrections in two syllables that are misaligned with respect to each other. (**a**) Training models of two experimental birds in imitation task 2 (left, Audio 5, playbacks 1 and 2; right, Audio 6, playbacks 3 and 4; see Supplementary Table 2). Scale bars for sonograms are 100ms (x axis) and 2kHz (y axis). (**b**) The median pitch of consecutive renditions of syllable A (top) and C (bottom) in bird 1 and 2. Both pitch errors were successfully corrected. (**c**) Stack plots showing consecutive motif

renditions in birds 1 and 2; colors, pitch of syllables A/A^t and C/C^t (t= target pitch); grayscale, Wiener entropy in neighboring syllables (as in Fig. 2b). Sonograms at bottom and top show song at start and end points. Birds corrected pitch errors in syllable A and C before changing syntax (bird 1 did not change syntax at all; bird 2 matched the target syntax). (**d**) Fraction of pitch error correction (left) and time (days) to reach 50% pitch match (right) in syllables A and C across experimental birds. Black, individual birds (lines connect the two syllables in each bird); red, mean ± s.e.m; n=8.

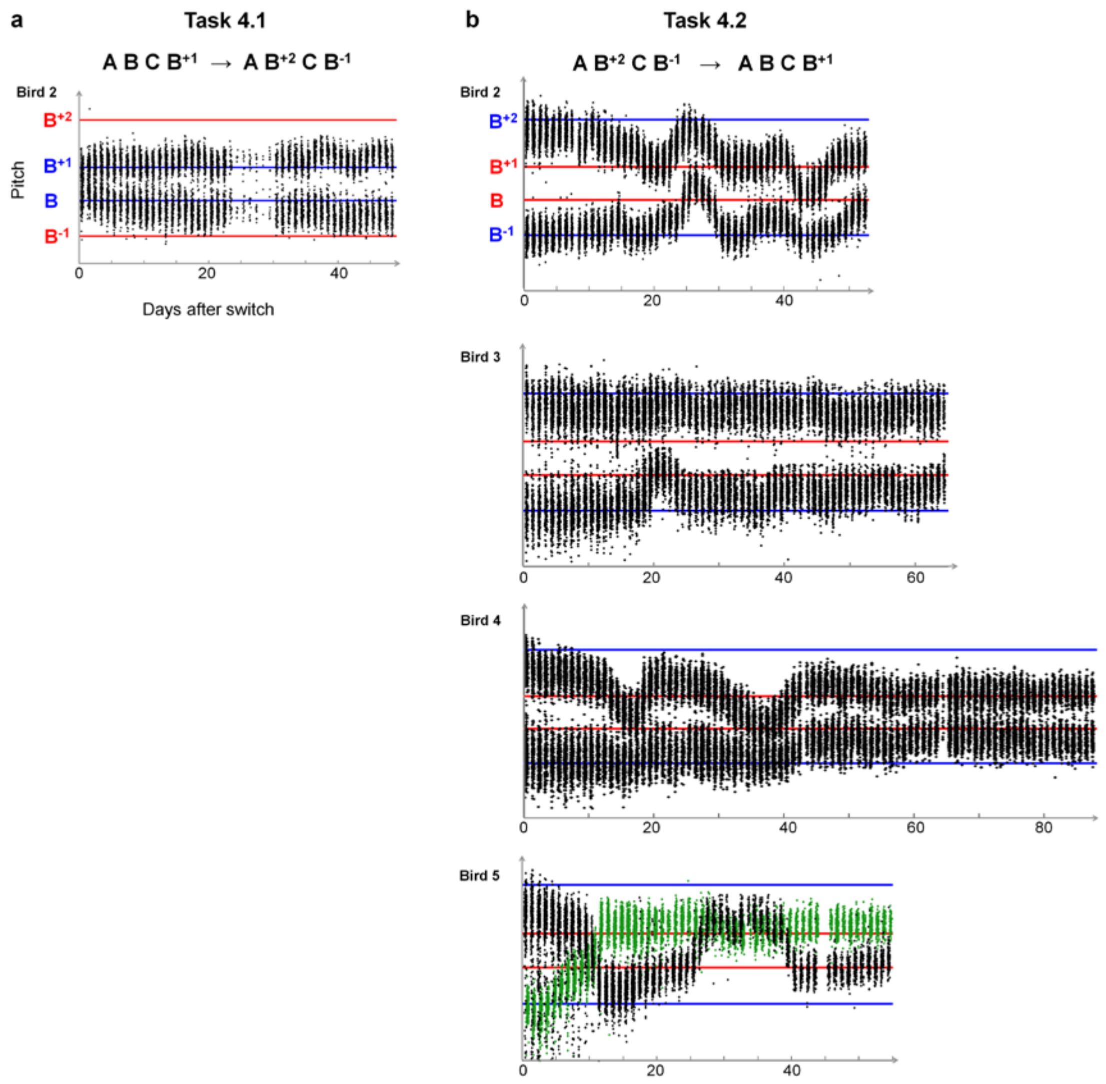

Supplementary Figure 2

Pitch trajectories in birds trained with tasks 4.1 and 4.2. Median pitch in consecutive renditions of the two pitch shifted syllable types in birds trained with task 4.1 **(**ABCB^{+1} → AB^{+2}CB^{-1}**)** (**a**) and task 4.2 **(**AB^{+2}CB^{-1} → ABCB^{+1}) (**b**), not including the two birds depicted in Fig 4c. Notation as in Fig 4c. In all birds except one (bird 5 in (**b**)), the pitch of both syllable types shifted towards the spectrally closer targets. In bird 5, the pitch of both syllable types (shown in black and green for visual clarity) shifted towards the spectrally farther targets. Bird ages at the end of the experimental period in task 4.2 were 121, 121, 128, 130 and 153 days post hatch. As the sensitive period for song learning in zebra finches ends around day 90-100 post hatch, it is unlikely that birds in this group that matched the 1-semitone targets were on the way to matching the farther targets.

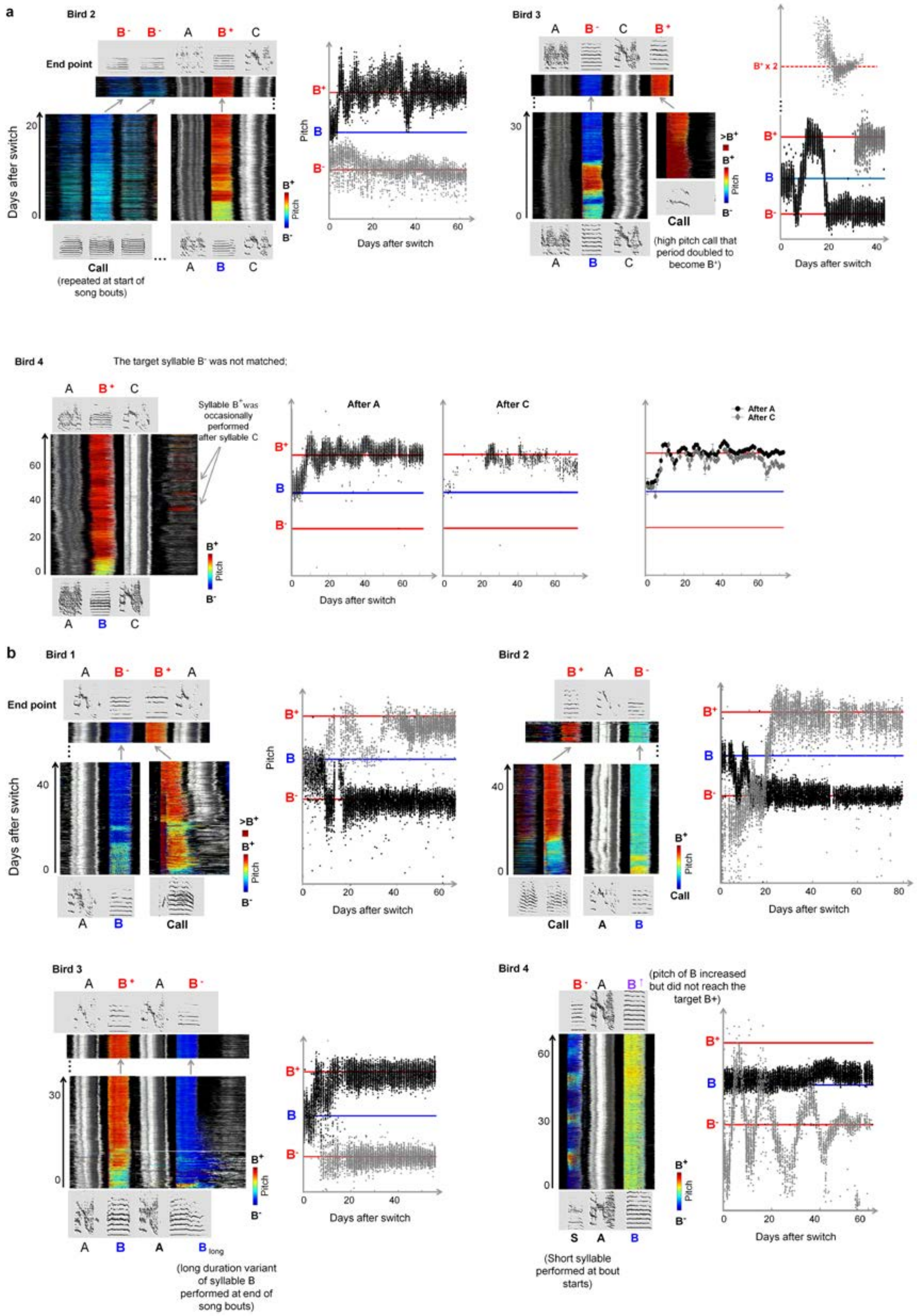

Supplementary Figure 3

Matching a vacant target with a vocalization initially performed outside of the song motif.

Developmental trajectories of experimental birds trained with tasks containing an extra syllable type in the target versus the source: task 3 **(**ABC → AB^{-}CB^{+}**)** (**a**) and

task 5 ($AB \rightarrow AB^{+}AB^{-}$) (**b**). Stack plots and pitch trajectories as in Fig 5 c and d (depicting Bird 1 of task 3). All birds except one (Bird 4 in (**a**)) matched the vacant target with a syllable type initially external to the song motif, usually a call. In bird 4 (**a**, bottom), the target B^{-} was not matched. Syllable B shifted to B^{+} in the “wrong” context (namely, after syllable A), but was also performed sparsely after syllable C; pitch trajectories in this bird are shown separately for renditions after A and after C (middle plots); right-most plot shows daily pitch means ± s.e.m. for renditions after A (black circles) and C (grey diamonds), showing a gradual divergence in pitch (756±1.6 Hz after A versus 731±1.9 after C on last experimental day; $p<0.00001$, ttest). This could potentially result from incomplete “splitting” of B^{+} into two syllable types.

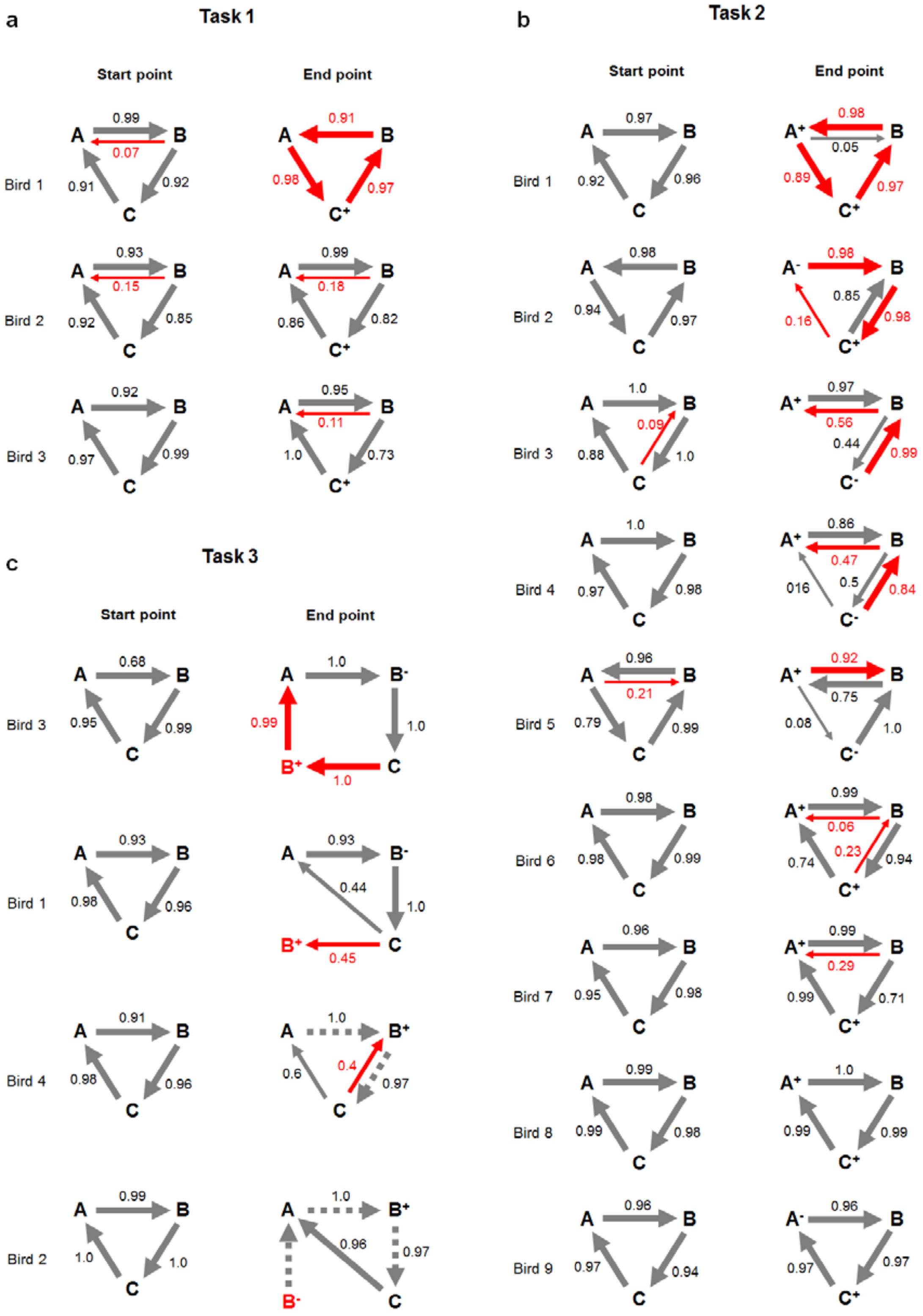
a
Task 1
Start point
End point
Bird 1
A B C 0.99 0.07 0.91 0.92
A B C+ 0.91 0.98 0.97
Bird 2
A B C 0.93 0.15 0.92 0.85
A B C+ 0.99 0.18 0.86 0.82
Bird 3
A B C 0.92 0.97 0.99
A B C+ 0.95 0.11 1.0 0.73
b
Task 2
Start point
End point
Bird 1
A B C 0.97 0.92 0.96
A+ B C+ 0.98 0.05 0.89 0.97
Bird 2
A B C 0.98 0.94 0.97
A- B C+ 0.98 0.85 0.16 0.98
Bird 3
A B C 1.0 0.09 0.88 1.0
A+ B C- 0.97 0.56 0.44 0.99
Bird 4
A B C 1.0 0.97 0.98
A+ B C- 0.86 0.47 016 0.5 0.84
Bird 5
A B C 0.96 0.21 0.79 0.99
A+ B C- 0.92 0.75 0.08 1.0
Bird 6
A B C 0.98 0.98 0.99
A+ B C+ 0.99 0.06 0.23 0.74 0.94
Bird 7
A B C 0.96 0.95 0.98
A+ B C+ 0.99 0.29 0.99 0.71
Bird 8
A B C 0.99 0.99 0.98
A+ B C+ 1.0 0.99 0.99
Bird 9
A B C 0.96 0.97 0.94
A- B C+ 0.96 0.97 0.97
c
Task 3
Start point
End point
Bird 3
A B C 0.68 0.95 0.99
A B- C B+ 1.0 0.99 1.0 1.0
Bird 1
A B C 0.93 0.98 0.96
A B- C B+ 0.93 0.44 1.0 0.45
Bird 4
A B C 0.91 0.98 0.96
A B+ C 1.0 0.6 0.4 0.97
Bird 2
A B C 0.99 1.0 1.0
A B+ C B- 1.0 0.96 0.97

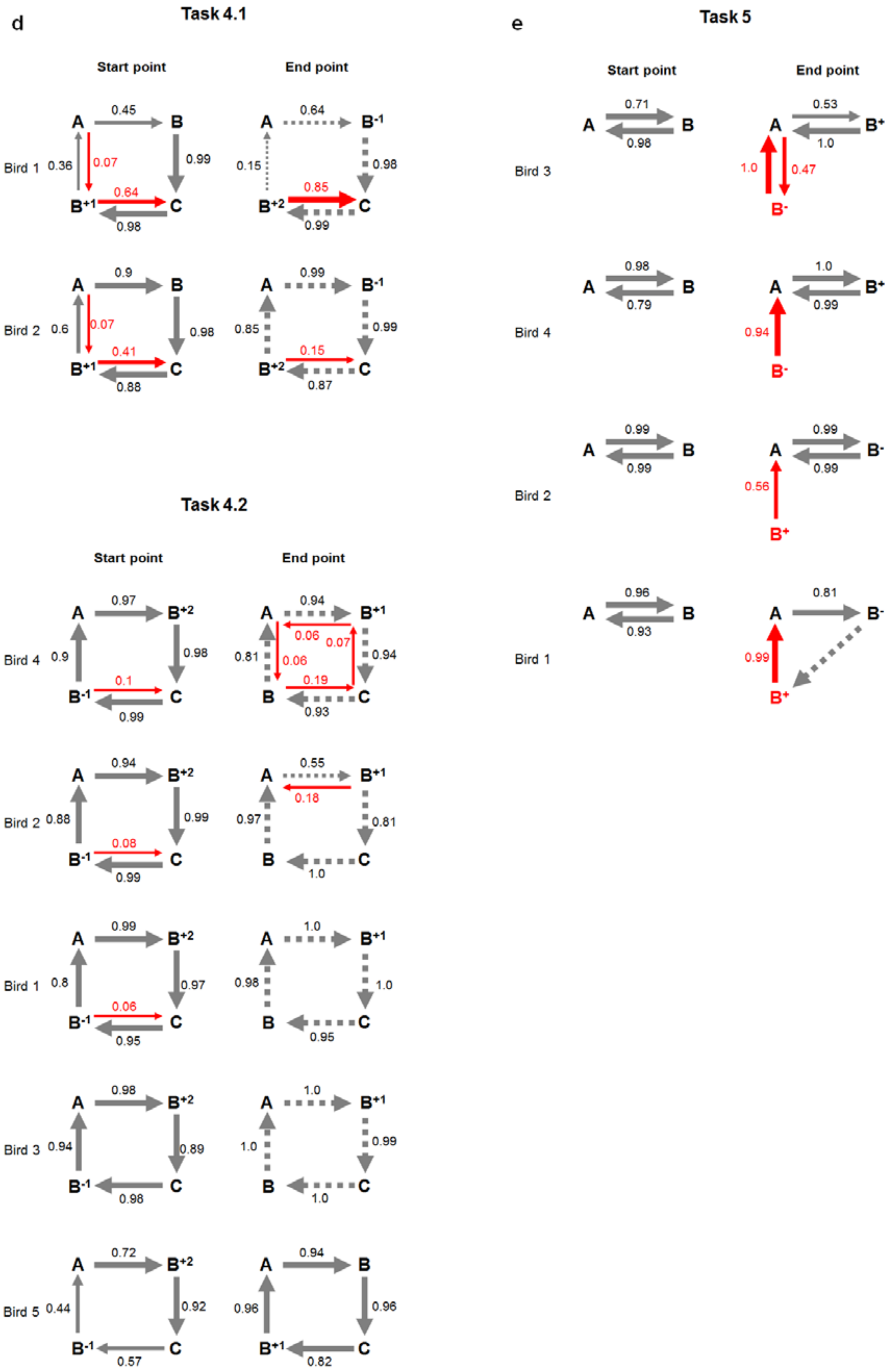
d
Task 4.1
Start point
End point
Bird 1
0.45
0.36
0.07
0.99
0.64
0.98
A
B
B+1
C
0.64
0.15
0.98
0.85
0.99
B-1
B+2
Bird 2
0.9
0.6
0.07
0.98
0.41
0.88
0.99
0.85
0.99
0.15
0.87
Task 4.2
Start point
End point
Bird 4
0.97
0.9
0.98
0.1
0.99
B+2
B-1
0.94
0.81
0.06
0.07
0.06
0.94
0.19
0.93
B+1
Bird 2
0.94
0.88
0.99
0.08
0.99
0.55
0.18
0.97
0.81
1.0
Bird 1
0.99
0.8
0.97
0.06
0.95
1.0
0.98
1.0
0.95
Bird 3
0.98
0.94
0.89
0.98
1.0
1.0
0.99
1.0
Bird 5
0.72
0.44
0.92
0.57
0.94
0.96
0.96
0.82
e
Task 5
Start point
End point
Bird 3
0.71
0.98
0.53
1.0
1.0
0.47
B+
B-
Bird 4
0.98
0.79
1.0
0.99
0.94
Bird 2
0.99
0.99
0.99
0.99
0.56
Bird 1
0.96
0.93
0.81
0.99

Supplementary Figure 4

Syntax adjustments towards target across experimental groups. a-e, Syntax diagrams of all experimental birds (tasks 1-5) at the start point of the experiment (the day of switching to target training, left) and at its endpoint (last experimental day, right). Arrows represent the fractions of performing the syllable transitions of the source song (gray) and the target song (red). The thickness of arrows corresponds to the fraction values, which are also indicated next to the arrows. Birds' names are the same as in Supplementary Figs. 2 and 3; birds in each group are vertically arranged according to the amount of syntax adjustments (maximum adjustment on top). **c-d**, dotted gray arrows represent incorrect transitions resulting from shifting syllable pitch to misaligned target syllables (note that bird 5 in (**d)** shifted pitch to the aligned targets, and therefore no syntax adjustments were necessary). Newly generated syllables in tasks 3 and 5 (**c** and **e**) are shown in red. In two cases (Bird 2 in **c** and Bird 1 in **e**) the new syllable was placed in an incorrect context in the song motif, indicated by a dotted gray arrow.

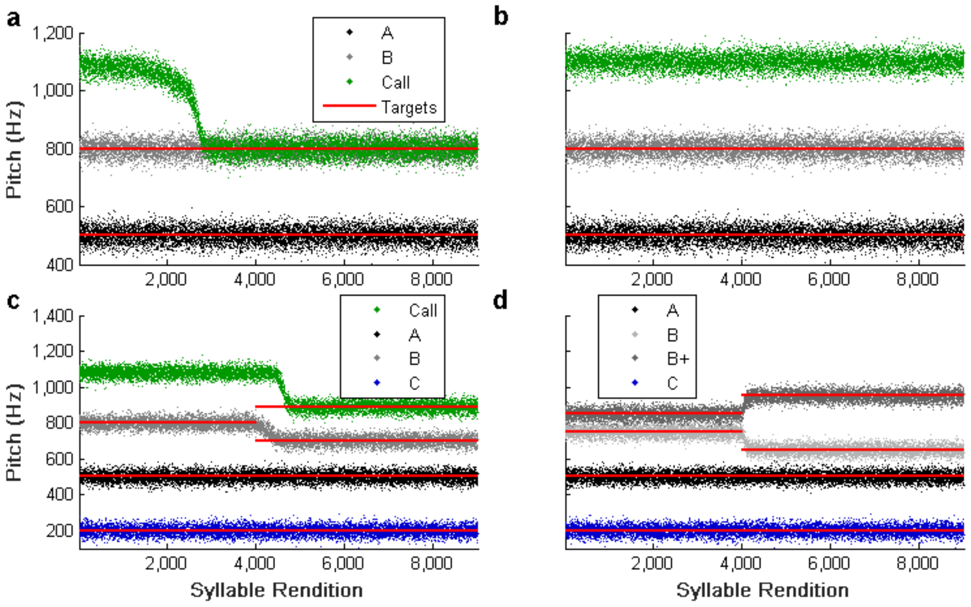

Supplementary Figure 5

Birds allocate vocalizations to models more sparsely than the EM algorithm. (**a**) A bird singing two-syllable-song with syllable A (black dots) and B (grey dots) matching two targets (red lines). The unassigned call (green dots) converges to the occupied target B in case of the standard EM algorithm (without Step $N3$), but (**b**) in case of the musical-chairs enhanced EM algorithm (including Step $N3$) in which posterior probabilities can be large for at most one syllable, the call is not attracted towards an occupied target. (**c**) Simulation of the $ABC \rightarrow AB^{-}CB^{+}$ task. After switching to the new targets (discontinuity in red lines) syllable B's pitch (grey dots) moves almost instantly to B^{-} and the pitch of the (initially) unassigned call (green) moves gradually to B^{+} (top red line). Syllable A (black dots) and syllable C (blue dots) stay on their targets. (**d**) Simulation of the $ABCB^{+1} \rightarrow AB^{+2}CB^{-1}$ task. After switching to the new targets the pitches of syllables B and B^{+} shift greedily to the closest targets, i.e. $B \rightarrow B^{-1}$ (lighter grey) and $B^{+1} \rightarrow B^{+2}$ (darker grey). Colored dots represent individual syllable renditions. The simulation parameters $(\alpha = 0.02, \alpha_2 = 0.5, \sigma = 25, \sigma_b = 75)$ are identical in all simulations.

Supplementary Table 1. Training models used in imitation task 1.

n	Source model (baseline pitch of syllable C, Hz)	Target model (superscripts, pitch shift from baseline, semitones)	Sound file
1	A B C(680) 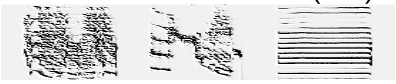	A C^{+2} B 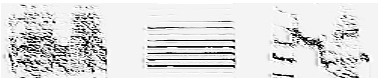	Audio 1 (playbacks 1 and 2)
2	A B C(680) 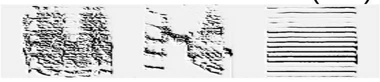	A C^{+1} B 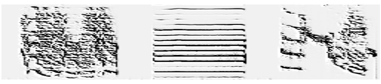	Audio 1 (playbacks 3 and 4)

Each model playback contained two repetitions of the motif shown (Audio 1; same for Supplementary Tables 2-5).

Supplementary Table 2. Training models used in imitation task 2.

n	Source model<br>(baseline pitch of syllables A and C, Hz)	Target model<br>(superscripts, pitch shift from baseline, semitones)	Sound file
2	**A**(1166) **B** **C**(680)<br>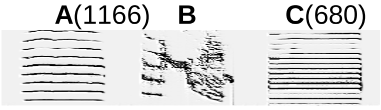	**A**$^{+2}$ **C**$^{+2}$ **B**<br>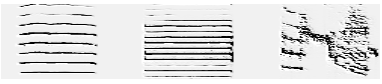	Audio 5 (playbacks 1 and 2)
2	**A**(1166) **B** **C**(680)<br>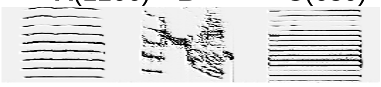	**A**$^{+1}$ **C**$^{+1}$ **B**<br>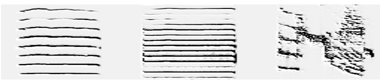	Audio 5 (playbacks 3 and 4)
2	**A**(1166) **B** **C**(680)<br>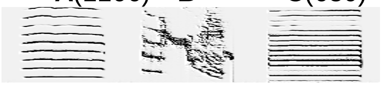	**A**$^{+1}$ **C**$^{-1}$ **B**<br>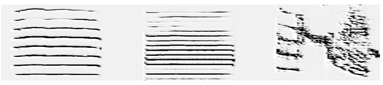	Audio 6 (playbacks 1 and 2)
1	**A**(1235) **C**(640) **B**<br>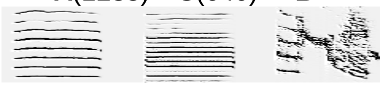	**A**$^{-2}$ **B** **C**$^{+2}$<br>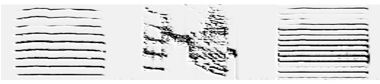	Audio 6 (playbacks 3 and 4)
1	**A**(1166) **B** **C**(680)<br>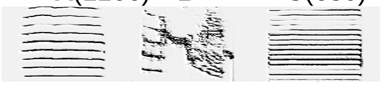	**B** **A**$^{-2}$ **C**$^{+2}$<br>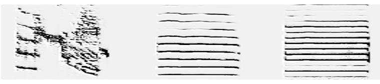	Audio 7 (playbacks 1 and 2)
1	**B** **A**(1042) **C**(762)<br>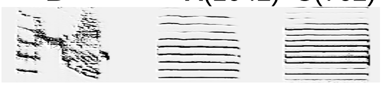	**B** **C**$^{-2}$ **A**$^{+2}$<br>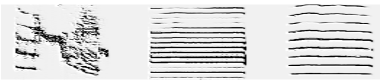	Audio 7 (playbacks 3 and 4)

Supplementary Table 3. Training models used in imitation task 3.

n	Source model (baseline pitch of syllable B, Hz)	Target model (superscripts, pitch shift from baseline, semitones)	Sound file
3	**A** **B**(680) **C** 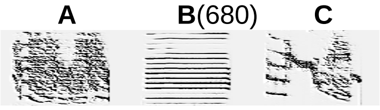	**A** $\mathbf{B}^{-2}$ **C** $\mathbf{B}^{+2}$ 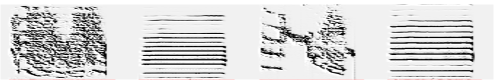	Audio 8 (playbacks 1 and 2)
1	**A** **B**(1166) **C** 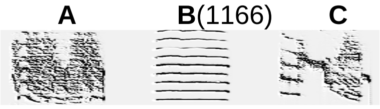	**A** $\mathbf{B}^{-2}$ **C** $\mathbf{B}^{+2}$ 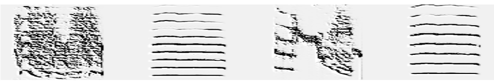	Audio 8 (playbacks 3 and 4)

Supplementary Table 4. Training models used in imitation task 4.

n	Source model	Target model	Sound file
2	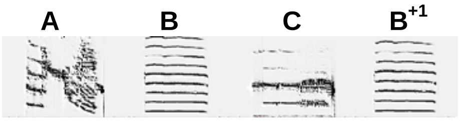	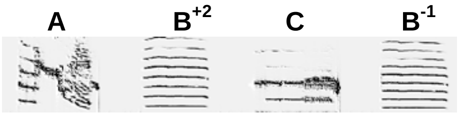	Audio 9 (playbacks 1 and 2)
1	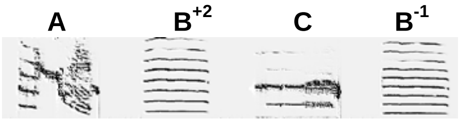	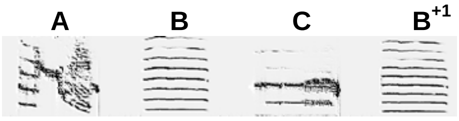	Audio 9 (playbacks 3 and 4)
4	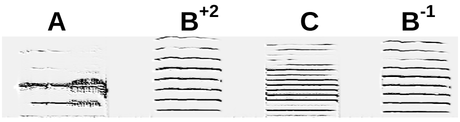	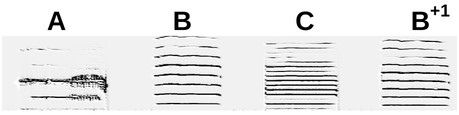	Audio 9 (playbacks 5 and 6)

All indicated pitch shifts are with respect to the baseline pitch of syllable B (1166 Hz).

Supplementary Table 5. Training models used in imitation task 5.

n	Source model (baseline pitch of syllable B, Hz)	Target model (pitch shift from baseline, semitones)	Sound file
4	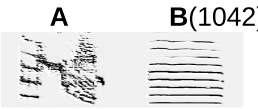	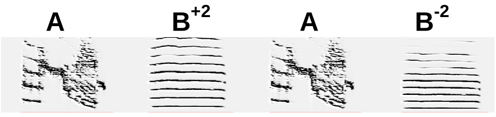	Audio 10

To avoid a large duration difference between source and target playbacks, in this task the source playbacks included 4 motif repetitions (Audio 10).

Supplementary Notes

Mathematical Supplement:

Birds simplify the quadratic problem of computing performance error to a linear assignment problem

Performance error

Associated with a song S we define a family of performance errors $E(S,\Delta)$ parameterized by a set of unknown parameters grouped in the matrix $\Delta = (\delta_{i,j})$. The errors are composed of an overall **phonology** (spectral) error and a **syntax** (sequence) error

$$E(S,\Delta) = \underbrace{\sum\nolimits_{i,j} e(S_j, T_i)\ \delta_{i,j}}_{\text{phonology}} + \underbrace{c\|\Delta\|_{\#}}_{\text{syntax}} \qquad (1)$$

The parameter c represents an unknown tradeoff between phonology and syntax errors.

The overall **phonology error** between song S and target T is defined as a weighted sum of the local phonological errors $e(S_j, T_i)$ between song element S_j (e.g. syllable $j = 1,\ldots,n$) and target element T_i ($i = 1,\ldots,m$). The function e represents a distance metric, for example the Euclidean distance between specific sound features such as pitch (e.g. pitch deviation). The unknown assignment matrix $\Delta = (\delta_{i,j})$ specifies the weight $\delta_{i,j}$ associated with the local phonology error, where target assignments (which syllables are assigned to a specific target) correspond to rows of Δ and syllable assignments (which targets are assigned to a specific syllable) to columns of Δ. To illustrate this notation, a bird that does not assign a phonology error to syllable S_2 entails $\delta_{i,2} = 0$ for all i; and, a bird that compares S_2 with the first target (syllable) T_1 entails $\delta_{1,2} = 1$. If there is local chaining of assignments then a bird that compares S_1 to T_3 will also compare S_2 to T_4; in terms of Δ, chaining of assignments means that the condition $\delta_{i,j} = 1$ implies $\delta_{i+1,j+1} = 1$ with high probability. By virtue of the assignment weights $\delta_{i,j}$, the phonology errors may parameterize any imaginable comparison between song and target.

The **syntax error** $\|\Delta\|_{\#}$ quantifies the amount of resequencing a bird must perform in order to bring its song elements into global alignment with the template. This error quantifies the new transitions to be created among existing song elements. Because of stepwise acquisition of syntax in songbirds[1], it makes sense to attribute to $\|\Delta\|_{\#}$ a cost proportional to the number of new transitions to be generated.

In the case of binary and one-to-one syllable-target assignments ($\delta_{i,j} = 0$ or 1; up to one target per syllable, $\sum_i \delta_{i,j} \leq 1, \forall j$, and one syllable per target,

$\sum_j \delta_{i,j} = 1, \forall i$) we can write $\|\Delta\|_\#$ as a sum of terms that are quadratic in the assignment weights $\delta_{i,j}$:

$$\|\Delta\|_\# = \sum_{i,j}^{m,n} \sum_{k \neq \lfloor j+1 \rfloor_n}^{n} \delta_{i,j} \delta_{\lfloor i+1 \rfloor_m, k} \tag{2}$$

where we use the short-hand notation $\lfloor x \rfloor_l$ to denote $1 + (x-1) \bmod l$, which is necessary to incorporate the circular boundary conditions arising from birds' tendency to repeat motifs several times in a song bout. As can be seen, Equation (2) skips all pairs of consecutive syllable assignments j and $\lfloor j+1 \rfloor_n$ that do not need resequencing, namely all those that are locally diagonally chained.

Equations (1) and (2) model the performance error attributed to any given song. The assignment matrix is not specified therein; therefore, these equations can be thought of as the space of all possible strategies for estimating phonology and syntax errors between song and template (Fig. 1a). Our experiments were designed to resolve birds' strategy in dealing with phonology and syntax errors and the on/off-diagonal structure of the assignment matrix.

Next we describe the process of song learning. In terms of Equation (1), song learning is the search of a song S with vanishing performance error.

Song learning

Song learning is the process of changing the current song S towards the **final song** S^* that minimizes the performance error (ideally $S^* = T$):

$$S^* = \arg\min_S E(S, \Delta) \tag{3}$$

In the process of song learning, Δ is either fixed or it evolves in time, possibly giving rise to very complex learning trajectories. If birds want to perform song learning optimally, they will try to compute the initially **optimal assignments** Δ^*, which are the ones that achieve minimal initial performance error,

$$\Delta^* = \arg\min_\Delta E(S, \Delta). \tag{4}$$

This optimal choice of assignment in Equation (4) is a quadratic assignment problem (quadratic in the assignment weights)[2]. In the general case, that problem is NP-hard, meaning that there is no known algorithm for solving this problem in polynomial time. Moreover, it was proven that such problems do not even have an approximation algorithm running in polynomial time[3]. Hence, almost certainly, birds neither solve Equation (4) nor an approximation thereof. The question for us was how birds actually assign performance errors.

Whatever birds do, we imagined they must be facing a tradeoff between phonology and syntax errors, illustrated by the following example: Consider two birds that need to change their songs from syllable sequence $ABC\ ABC$ to $ACB\ ACB$. The first bird forms 3 new bigrams (AC, CB, and BA) among the existing

syllables, which would imply that its syntax error initially is $c\left\| \begin{matrix} 1 & 0 & 0 \\ 0 & 0 & 1 \\ 0 & 1 & 0 \end{matrix} \right\|_{\#} = 3c$ and the phonology error is zero. However, the second bird achieves the same target song not by permuting syllables but by making the local transformations $B \to C$ and $C \to B$, implying that this latter bird produces no syntax errors $\left\| \begin{matrix} 1 & 0 & 0 \\ 0 & 1 & 0 \\ 0 & 0 & 1 \end{matrix} \right\|_{\#} = 0$ but a phonology error of $2e(B,C)$. This example illustrates that there is a tradeoff between phonology and syntax errors and that the latter are avoidable in principle. The reason is that the second bird learns the song sequence intrinsically by globally aligning the song to the template (Δ is the identity matrix), that latter bird needs only to correct phonology errors to also automatically learn the correct syntax. However, global alignment may not be an ideal strategy because it can entail a high phonology cost, which is absent in the first bird. As a tradeoff we imagine that birds may chain alignments locally rather than globally. Such chaining is for example suggested by the sequence requirement for correct acoustic models in white-crowned sparrows[4].

<u>Experimental characterization of Δ</u>

Our experiments provide the following constraints $C1$ to $C4$ on error assignments in zebra finches:

$C1$) $ABC \to AC^{+}B$ and $ABC \to A^{+}C^{+}B$ (imitation tasks 1 and 2): Birds perform the local change $C \to C^{+}$; therefore, off-diagonal elements of Δ can be nonzero (**no global alignment**).

$C2$) $ABC \to AB^{-}CB^{+}$ (imitation task 3): Birds do not chain locally: as target for B, they select either B^{-} (in context) or B^{+} (out of context). Because birds do not choose an interpolation between B^{-} and B^{+} as target, it follows that in each column of Δ at most one assignment weight is nonzero, i.e., $\delta_{i,j} = \{0,1\}$ (**selection**); and $\sum_i \delta_{i,j} \le 1, \forall j$ (**winner-takes-all**).

$C3$) $ABCB^{+1} \to AB^{+2}CB^{-1}$ (imitation task 4): Birds make the changes $B \to B^{-1}$ and $B^{+1} \to B^{+2}$ implying that assignments are **greedy**: $\delta_{i,j} = 1$ in syllable-target pairs for which the local phonology error $e(S_j, T_i)$ is minimal, and $\delta_{i,j} = 0$ otherwise.

$C4$) In all experiments, no two syllables or calls converge on the same target (**musical chairs**); therefore, in each row of Δ exactly one assignment weight is nonzero, i.e. $\sum_j \delta_{i,j} = 1, \forall i$.

Linear assignment problem

In combination, our observations show that birds greedily choose a (binary) assignment matrix Δ^* associated with minimal phonology error:

$$\Delta^* = \arg\min_\Delta \sum_{i,j} \left\| S_j - T_i \right\| \delta_{i,j} \qquad (5)$$

where $e(S_j, T_i) = \left\| S_j - T_i \right\|$ is the absolute pitch difference between syllable j and target i, and where Δ is a $m \times n$ permutation matrix with at most one nonzero entry per column and exactly one nonzero entry per row ($\delta_{i,j} = \{0,1\}$, $\sum_i \delta_{i,j} \le 1, \forall j$, and $\sum_j \delta_{i,j} = 1, \forall i$). Equation (5) fully specifies the assignment matrix; what is particularly interesting is that the optimization in Equation (5) does not depend on the tradeoff constant c, implying absence of a tradeoff. The optimization in Equation (5) is known as the linear assignment problem which can be conveniently solved using for example the Hungarian method[5].

In the context of natural language processing, the solution to Equation (5) (the minimum in Equation (5) rather than its argument) is also known as the word mover's distance[6] that represents the distance between two text documents. In that analogy, $\left\| S_j - T_i \right\|$ represents the distance between an individual word S_j in a source document and a word T_i in a target document. The assignments $\delta_{i,j}$ (which do not have to be binary but can take arbitrary nonnegative values), represent the flows between words. These flows have to sum up to match the bag-of-words (vocabulary) representations d_i and $d_j^{'}$ of the source and target documents, $\sum_j \delta_{i,j} = d_i$ and $\sum_i \delta_{i,j} = d_j^{'}$, in analogy to the musical chairs competition we find. The word mover's distance outperforms other approaches on many benchmark document categorization tasks[6,7].

The fact that birds choose the assignment of minimal overall phonology error, irrespective of syntax, demonstrates a radical way of dealing with the intractability of the general assignment problem. Namely, rather than getting entangled with high complexity and large cognitive demand, birds decide to solve a much simpler tractable problem and do this remarkably well.

The surprising implications are that birds do not consider the cost of resequencing at all when correcting phonology errors. Phonology errors seem to be associated with a high cost, perhaps reflecting the amount of effort required to change syllable pitch. Counterintuitively, birds behave in this process as if there were no resequencing cost at all, despite the fact that this cost is seemingly very high, given that most birds try to re-sequence their syllable strings but only few succeed. Namely, we found that many birds do not reach the global performance error minimum in Equation (3) but get stuck somewhere on the way where some syntax errors but usually no phonology errors remain.

In summary, song learning is a modular, two-fold process. In a first process, birds choose assignments Δ^* by solving a linear problem based on their vocal repertoire but not on their song sequence. In the second process, birds reduce

phonology errors defined by these correspondences and independently and more slowly, also reduce the resulting syntax error.

Sub-syllabic notes

What is the smallest song unit to which our formalism applies? We deliberately called S_j a song element and T_i a target element, implying these elements do not necessarily have to represent entire song syllables but could also represent sub-syllabic notes. In the following we discuss this possibility.

In our treatment of the song learning problem, we implicitly assumed that birds compute phonological error of a syllable by integrating over the errors in its constituent notes. Essentially, we assumed that birds compute the error of a syllable by globally aligning its notes with that of a template syllable. However, we have no evidence for this mini-version of global alignment. Thus, it remains to be explored whether birds can assign one of its syllable notes either to a note in a different syllable of the template or to a note in a different position within the same template syllable.

Although it will not be possible to resolve this issue without further experimenting, we imagine that our discovered assignment strategy cannot apply to ever smaller song units. Namely, at some point, there must be an overload to short-term memory arising from all these pairwise comparisons between song and template elements. It is therefore likely that assignment capabilities of zebra finches are limited to the syllable level and do not generalize to smaller song units below that level.

How to match syllable vocabulary using the expectation maximization (EM) algorithm and Gaussian mixture models

Song learning can be considered a density estimation problem in which the unknown parameters of the developing song syllables must be identified such that good matches with the sensory targets are achieved. In a Gaussian mixture model, the observable data points T_i (renditions of the $i=1,\dots,m$ target syllables) are modeled as a superposition of n Gaussian probability densities, where n is the number of distinct song syllables and calls in the juvenile's repertoire. In the one dimensional case of fitting the pitch parameter alone, the distribution of pitch X_j of syllable j $(j=1,\dots,n)$ is given by $P(X_j)=\frac{1}{\sigma\sqrt{2\pi}}e^{-\frac{(X_j-S_j)^2}{2\sigma^2}}$, where S_j is the mean pitch of that syllable. It follows that the likelihood density that syllable j will produce the pitch T_i of template i is given by

$$P_{i|j}=\frac{1}{\sigma\sqrt{2\pi}}e^{-\frac{(T_i-S_j)^2}{2\sigma^2}}. \tag{6}$$

Here we assume that σ^2 is the constant pitch variance of syllable j. The closer the mean pitch S_j is to the target pitch T_i, the more likely the production of syllable j will match the target i.

The goal of the EM algorithm is to identify the set of mean syllable pitches S_j that maximize the total probability P (or its logarithm) of reproducing all the target pitches T_i. The following function L is usually maximized by the EM algorithm: $L=\sum_i \ln(P_i)=\sum_i \ln\left(\sum_j P_{i|j}p_j\right)$, where $P_i=\sum_j P_{i|j}p_j$ is the probability that target i is produced by any of the n syllables, p_j being the prior probability of singing syllable j.

In the following, we assume that all syllables have identical prior probability, $p_j=p$ (zebra finches sing motifs with linear syllable arrangement) and drop any further mention of p because it is an irrelevant constant. Note that the EM algorithm is an iterative algorithm aimed not at directly maximizing L, but rather its lower bound $L_L<L$:

$$L_L=\sum_{i,j} P_{j|i}\ln\left(P_{i|j}\right), \tag{7}$$

where $P_{j|i}$ is referred to as the posterior probability (that syllable j is assigned to target i). To avoid confusing the posterior probability $P_{j|i}$ with the Gaussian likelihood $P_{i|j}$, it is always assumed that the index j refers to a syllable and index i refers to a target. The maximization of L_L in Equation (7) is usually done in two steps, an E step in which the posterior probabilities are updated:

$$P_{j|i} = \frac{P_{i|j}}{\sum_j P_{i|j}}, \quad (8)$$

and an M step in which the mean pitches are updated according to

$$S_j = \frac{\sum_i T_i P_{j|i}}{\sum_i P_{j|i}}. \quad (9)$$

Back-and-forth iteration of Equations (8) and (9) usually leads to convergence of the set of mean pitches S_j towards the set of targets T_i.

Birds' strategy compared with the EM algorithm

The EM algorithm operating on Gaussian mixture models just outlined exhibits several similarities with birds' strategy of minimizing performance error. Namely, if we place the Gaussian model in Equation (6) into the function L_L to be maximized in Equation (7), then we obtain the following expression:

$$L_L = K - \frac{1}{2\sigma^2}\sum_{i,j} P_{j|i}\left(T_i - S_j\right)^2 , \quad (10)$$

with $K = -m\ln\left(\sigma\sqrt{2\pi}\right)$ being a constant without relevance for the maximization (because σ is assumed to be constant). The maximization in Equation (7) is identical with the minimization in Equation (5), provided we interpret the posterior probabilities $P_{j|i}$ as assignment weights $\delta_{i,j}$.

In the EM algorithm, the posterior probabilities $P_{j|i}$ are not constrained to be binary variables that take values either zero or one. Nevertheless, the E and M steps in Equations (8) and (9) achieve to a good approximation the musical chairs competition we found.

To see this, consider the case in which for a given target there is only a single syllable with similar pitch ($P_{i|j}$ is large only for a single syllable j). According to Equation (8), $P_{j|i}$ is close to 1 for that best matching syllable j and close to 0 for the other, non-matching syllables. This means that Equation (8) implements a soft competition among syllables ($\sum_j \delta_{i,j} = 1$), which is an approximation of the musical chairs interactions among syllables we found.

In a similar way, the normalization in Equation (9) implements a soft winner-takes-all mechanism. Namely, if for a given syllable j one of the posterior probabilities (assignment weights) $P_{j|i}$ is large and the other very small, then by the weighted sum in Equation (9), that syllable's pitch is drawn towards the pitch of the assigned target.

To model the slow and gradual song development seen in birds, we simulated a finely discretized version of the EM algorithm in Equations (8) and (9). Because birds change pitch continuously and slowly unlike in Equations (8) and (9), we implemented a slow dynamical system in which we replaced the possibly large and discontinuous posterior probability and mean pitch changes in Equations (8) and (9) by gradual iterative processes (iterating over renditions t):

1) We replaced the mean pitch S_j defined in Equation (9) by the iterative variables

$$S_j^{t+1} = S_j^t + \alpha\left(\sum_i T_i P_{j|i} - S_j^t \sum_i P_{j|i}\right), \quad (11)$$

where the index t labels the rendition number of the syllable, and α is a small integration rate. At a steady state S_j^t equals S_j.

2) We sampled the pitch X_j^t of the rendition t of syllable j according to the Gaussian model

$$P\left(X_j^t\right) = \frac{1}{\sigma\sqrt{2\pi}} e^{-\frac{\left(X_j^t - S_j^t\right)^2}{2\sigma^2}}. \quad (12)$$

3) We assume that birds can compute random samples and estimate their density, but they cannot explicitly compute probabilities. We thus sampled the likelihoods $P_{i|j}$ in Equation (6) via random variables b_{ij}^t and their running averages n_{ij}^t. First, we computed the instantaneous likelihood as a binary random variable b_{ij}^t:

$$\mathrm{P}\left(b_{ij}^t = 1\right) = \frac{1}{\sigma_b\sqrt{2\pi}} e^{-\frac{\left(T_j - X_j^t\right)^2}{2\sigma_b^2}} \quad (13)$$

and $b_{ij}^t = 0$ otherwise with parameter σ_b. Second, we estimated the posterior probabilities in Equation (8) according to $P_{j|i}^t = \frac{n_{ij}^t}{\varepsilon + \sum_l n_{il}^t}$ with ε a small regularization constant and n_{ij}^t a running average of b_{ij}^t:

$$n_{ij}^{t+1} = \left(1 - \alpha_2\right) n_{ij}^t + \alpha_2 b_{ij}^t \quad (14)$$

with integration rate α_2.

We simulated birds that produced three syllables ($n = 3$) and had to match three targets ($m = 3$). Therefore, we iterated back and forth the above expressions for S_j^t and $P_{j|i}^t$.

In simulations, we realized that the musical chairs competition is not well captured by these equations: A call that did not match any target tended to converge to a nearby target regardless whether the latter was occupied or not (Supplementary Fig. 5a). To remedy this discrepancy, we hardened the musical chairs competition by adding the constraint that at each sampled likelihood, b_{ij} could be 1 for at most one vocalization, implying that matched targets could not attract any unassigned syllables or calls (Supplementary Fig. 5b). We achieved this constraint by setting b_{ij} to zero for all j whenever for a given target i two or more b_{ij}'s were sampled to be one (i.e., when $\sum_j b_{ij} > 1$ we set $b_{ik} = 0,\ \forall k$).

In summary, we iteratively simulated the system in 5 steps ($N1$ to $N5$):

$N1$: sample pitch X_j^t according to Equation (12)

$N2$: sample the instantaneous likelihood b_{ij}^t according to Equation (13)

$N3$: enforce musical chairs: $\forall i,$ if $\sum_j b_{ij}^t > 1$ set $b_{ik}^t = 0 \ \forall k$

$N4$: update running average according to Equation (14)

$N5$: update mean pitch according to Equation (11)

The syllable trajectories X_j^t resulting from running this system are shown in Supplementary Fig. 5b-d.

The interesting property of these equations is that they in essence capture the observations without requiring any parameter fitting other than σ_b. The latter was set to exceed the pitch standard deviation σ. This allowed for medium size pitch shifts of two semitones. The integration rates α and α_2 dictates the speed of pitch shifting, these parameters are set to yield smooth looking transitions.

In summary, the competition we find in birds is harder than that in the standard EM algorithm, in the sense that the EM algorithm brings all Gaussian models (syllables) to observables (targets), even if there are just 2 targets and 3 models, very unlike birds that bring only one syllable or call to each target, in a presumed attempt to limit used syllable resources. In a sense, birds are more efficient than the traditional EM algorithm, similarly to ongoing machine-learning approaches for restricting the effective number of model parameters to prevent overfitting, such as sparse priors[8,9], Bayesian learning, and Dirichlet processes[10,11]. We believe that greedy and competitive error assignment during vocal learning illustrates the importance of minimizing used resources.

Supplementary References

1. Lipkind, D. *et al.* Stepwise acquisition of vocal combinatorial capacity in songbirds and human infants. *Nature* **498,** 104–8 (2013).
2. Koopmans, T. C. & Beckmann, M. J. Assignment Problems and the Location of Economic Activities Author (s): Tjalling C . Koopmans and Martin Beckmann. *Econometrica* **25,** 53–76 (1957).
3. Sahni, S. & Gonzalez, T. P-Complete Approximation Problems. *J. ACM* **23,** 555–565 (1976).
4. Rose, G. J. *et al.* Species-typical songs in white-crowned sparrows tutored with only phrase pairs. *Nature* **432,** 753–8 (2004).
5. Kuhn, H. W. The Hungarian method for the assignment problem. *Nav. Res. Logist. Q.* **2,** 83–97 (1955).
6. Kusner, M. J., Sun, Y., Kolkin, N. I. & Weinberger, K. Q. From Word Embeddings To Document Distances. *Proc. 32nd Int. Conf. Mach. Learn.* (2015).
7. Huang, G., Guo, C., Kusner, M., Sun, Y. & Sha, F. Supervised Word Mover's Distance. *Adv. Neural* (2016).
8. Olshausen, B. A. & Field, D. J. Sparse coding with an overcomplete basis set: A strategy employed by V1? *Vision Res.* **37,** 3311–3325 (1997).
9. Tipping, M. Sparse Bayesian learning and the relevance vector machine. *J. Mach. Learn. Res.* **1,** 211–244 (2001).
10. Neal, R. M. *Bayesian Learning for Neural Networks*. (Springer Science & Business Media, 2012).
11. Ferguson, T. A Bayesian analysis of some nonparametric problems. *Ann. Stat.* (1973).
