# Supplementary material for: Songbirds work around computational complexity by learning song vocabulary independently of sequence
Source: Nat Commun. 2017 Nov 1;8:1247. doi: 10.1038/s41467-017-01436-0 (PMC5663719; doi:10.1038/s41467-017-01436-0)
Supplement: Supplementary file 3 — Description of Additional Supplementary Information [file 41467_2017_1436_MOESM3_ESM.pdf]

Description of Additional Supplementary Files

File Name: Supplementary Movie 1
Description: Training models used in Task 1 (See Supplementary Table 1) Playbacks are separated by 2 seconds of silence (same for Audio 5-10).

File Name: Supplementary Movie 2
Description: Example song bouts of an experimental bird trained with Task 1 (A B C → A C+2 B; Supplementary Table 1, first row; Fig. 2b-c) at different developmental stages; switch day (age 65 dph).

File Name: Supplementary Movie 3
Description: Example song bouts of an experimental bird trained with Task 1 (A B C → A C+2 B; Supplementary Table 1, first row; Fig. 2b-c) at different developmental stages; corrected pitch error, but not syntax error (age 70 dph).

File Name: Supplementary Movie 4
Description: Example song bouts of an experimental bird trained with Task 1 (A B C → A C+2 B; Supplementary Table 1, first row; Fig. 2b-c) at different developmental stages; learned target song (age 100 dph). Note that the bird begins each singing bout with one or more short introductory notes, and sings variable number of motif repetitions per bout (not necessarily two repetitions as in the model).

File Name: Supplementary Movie 5
Description: Training models used in Task 2 (See Supplementary Table 2).

File Name: Supplementary Movie 6
Description: Training models used in Task 2 (See Supplementary Table 2).

File Name: Supplementary Movie 7
Description: Training models used in Task 2 (See Supplementary Table 2).

File Name: Supplementary Movie 8
Description: Training models used in Task 3 (See Supplementary Table 3).

File Name: Supplementary Movie 9
Description: Training models used in Task 4 (See Supplementary Table 4).

File Name: Supplementary Movie 10
Description: Training models used in Task 5 (See Supplementary Table 5).
